# Research progress on the role and mechanism of STIM and Orai protein-mediated store-operated calcium entry in cardiovascular diseases

**DOI:** 10.1080/19336950.2026.2709185

**Published:** 2026-07-26

**Authors:** Xin Liao, Chenglin Pan, Xueqing Guo, Jun Cheng

**Affiliations:** Key Laboratory of Medical Electrophysiology of Ministry of Education and Medical Electrophysiological Key Laboratory of Sichuan Province, Collaborative Innovation Center for Prevention and Treatment of Cardiovascular Disease, Institute of Cardiovascular Research, Public Center of Experimental Technology, Hemodynamics and Medical Engineering Combination Key Laboratory of Luzhou, Southwest Medical University, Luzhou, China

**Keywords:** SOCE, STIM1, hypertension, cardiovascular disease

## Abstract

Store-operated calcium entry (SOCE) mediated by STIM and Orai proteins is a fundamental Ca^2+^ influx mechanism that critically regulates intracellular calcium homeostasis and participates in cardiovascular pathophysiology. Upon endoplasmic reticulum Ca^2+^ store depletion, STIM1/2 activate plasma membrane Orai1/3 channels, initiating Ca^2+^ entry that drives vasoconstriction, smooth muscle proliferation, platelet activation, and cardiac hypertrophy. Dysregulated SOCE is closely associated with hypertension, atherosclerosis, pulmonary hypertension, and thromboembolic disorders. However, SOCE is not a simple binary pathway but operates within a complex regulatory network. Beyond the core STIM-Orai axis, auxiliary proteins including transient receptor potential canonical 1 (TRPC1), tetraspanin 18 (Tspan18), tropomyosin 3 (TPM3), SOCE-associated regulatory factor (SARAF), and A-kinase anchoring protein 79/150 (AKAP79/150) modulate SOCE amplitude, kinetics, and downstream signaling in a cell- and context-dependent manner. Moreover, the functional consequences of SOCE are highly heterogeneous: Orai1 protects adult cardiomyocytes but promotes pathological hypertrophy in neonatal cells, posing a therapeutic dilemma. Although preclinical studies have shown efficacy of SOCE inhibitors, clinical translation remains hindered by poor isoform selectivity, suboptimal pharmacokinetics, lack of tissue-specific delivery, disease-stage-dependent effects, and absence of validated biomarkers. Importantly, recent evidence has definitively ruled out amlodipine-induced CRAC channel activation at therapeutic concentrations, confirming it as an experimental artifact. This review systematically summarizes the molecular complexity, functional diversity, and translational barriers of STIM/Orai-mediated SOCE, aiming to inform precision therapeutic strategies for cardiovascular diseases.

## Introduction

With the continuous advancement of modern medicine, public attention toward cardiovascular diseases has been increasingly growing. Cardiovascular diseases remain the leading cause of death globally, with projections indicating a 73.4% increase in crude mortality from 2025 to 2050 [1]. Although numerous therapeutic interventions are currently available, cardiovascular diseases continue to impose a substantial global burden, a trend that has become particularly evident following the COVID-19 pandemic [2]. Consequently, the exploration of novel therapeutic targets and signaling pathways is of paramount importance in contemporary cardiovascular research.

As a pivotal intracellular second messenger, Ca^2+^ plays a critical role in a multitude of physiological processes, including muscle contraction, neuronal transmission, and blood coagulation. It is an integral component of numerous signaling pathways that regulate essential cellular processes such as growth, pathogenesis, and cell death [3,4]. In recent years, the role of Ca^2+^ in cardiovascular diseases has become a research hotspot and has attracted considerable attention in the scientific community. Dysregulation of Ca^2+^ signaling can lead to disturbances in multiple intracellular signaling pathways. This, in turn, impairs or ablates specific cellular functions, and this cascade of events ultimately promotes the onset and progression of cardiovascular disease [5,6]. Therefore, the regulation of intracellular Ca^2+^ has become a prominent focus in recent medical research. In particular, the store‑operated calcium entry (SOCE) mechanism, also referred to as capacitative calcium entry , has attracted significant interest in the scientific community. Since Putney first introduced the concept of SOCE, persistent exploration and refinements by his own work and researchers worldwide have advanced the study of SOCE mechanisms to a relatively mature stage in the field of cellular signal transduction, with its functions and regulatory mechanisms being extensively investigated and validated [7–9]. There is a broad consensus that SOCE is a critical mechanism for intracellular calcium signaling, playing indispensable roles in a range of processes from maintaining normal cellular function and immune responses to the physiology and pathogenesis of cardiovascular diseases [10].

The canonical SOCE pathway is initiated upon activation of G protein-coupled receptors, leading to phospholipase C-mediated production of inositol 1,4,5-trisphosphate (IP_3_). IP_3_ binds to its receptor on the endoplasmic/sarcoplasmic reticulum (ER/SR), triggering Ca^2+^ release and store depletion [11]. This depletion is sensed by the ER-resident protein STIM1, which undergoes a conformational change, oligomerizes, and translocates to ER-plasma membrane junctions to directly gate and activate the Orai1 channel on the plasma membrane, mediating a sustained Ca^2+^ influx [12,13]. To prevent Ca^2+^ overload, SOCE is tightly regulated by negative feedback mechanisms, including fast and slow Ca^2+^‑dependent inactivation [14]. The STIM protein family comprises two isoforms, STIM1 and STIM2 [15], while the Orai family consists of Orai1, Orai2, and Orai3 [16]. These core components act in concert to orchestrate intracellular Ca^2+^ homeostasis.

However, emerging evidence has revolutionized our understanding of SOCE, revealing a regulatory complexity far beyond this classical binary pathway. At the molecular level, multiple splice variants of STIM2, such as the inhibitory STIM2.1, exert bidirectional modulation of Orai1-SOCE [17,18], while accessory proteins including TRPC1 [19], Tspan18 [20], and TPM3 [21] contribute to SOCE fine-tuning through distinct mechanisms involving channel composition, membrane trafficking, and protein–protein interactions. In this context, as systematically reviewed by Saint-Martin Willer et al., SOCE operates within a complex regulatory network where auxiliary proteins modulate its amplitude, kinetics, and downstream signaling in a cell‑type and context-dependent manner [22]. Moreover, Orai channels can also be activated in a store-independent manner, such as by leukotriene C_4_-mediated Orai1/3 heteromeric channels [23], further diversifying the functional repertoire of SOCE. At the pathophysiological level, the function of SOCE is not uniformly pathogenic; for instance, Orai1 exerts a protective role in adult cardiomyocytes while promoting hypertrophy and remodeling under specific pathological conditions [24], posing a significant challenge for therapeutic strategy design. In terms of clinical translation, SOCE inhibitors face multiple hurdles including insufficient isoform selectivity [25], suboptimal pharmacokinetics, lack of tissue-specific delivery systems, and the absence of validated biomarkers [26]. Of particular note, recent claims regarding amlodipine—a widely used first-line dihydropyridine L-type Ca^2+^ channel blocker for the treatment of hypertension-induced CRAC channel activation—have been conclusively demonstrated to be experimental artifacts arising from fluorescence interference, with no effect on SOCE at therapeutic concentrations [27,28].

Therefore, this review provides a systematic and critical overview of the STIM/Orai-mediated SOCE pathway in cardiovascular diseases. We focus on dissecting the molecular complexity of the SOCE regulatory network, the cell‑type dependent functional heterogeneity of its components, and the core translational bottlenecks that must be overcome. By integrating these perspectives, we aim to provide a theoretical foundation for the development of precision targeting strategies for this critical signaling axis in cardiovascular disease.

## STIM-Orai: Core components of CRAC channel function

### STIM protein family

**Figure 1.**
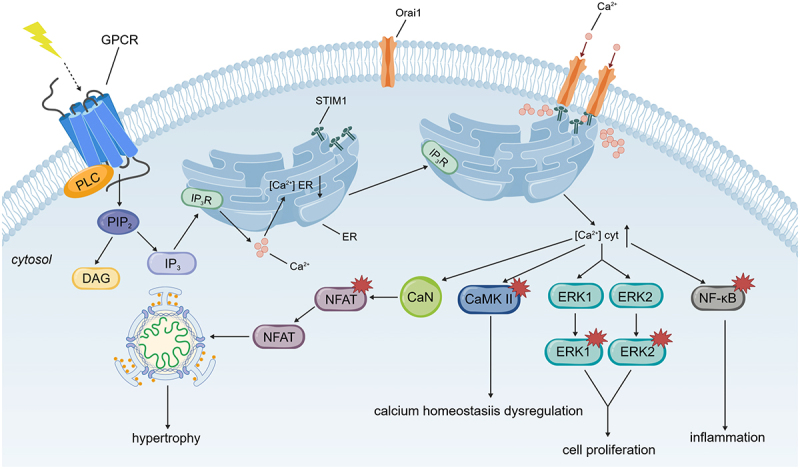
Schematic overview of the store-operated calcium entry (SOCE) pathway. Extracellular stimuli (e.g. agonists, hormones, or growth factors) bind to G protein-coupled receptors (GPCRs) or receptor tyrosine kinases, activating phospholipase C (PLC). PLC hydrolyzes phosphatidylinositol-4,5-bisphosphate (PIP_2_) to generate inositol-1,4,5-trisphosphate (IP_3_) and diacylglycerol (DAG). IP_3_ binds to IP_3_ receptors (IP_3_R) on the endoplasmic reticulum (ER), inducing Ca^2+^ release from the ER lumen into the cytoplasm and depleting ER Ca^2+^ stores. This reduction in ER Ca^2+^ concentration is sensed by the ER-resident Ca^2+^ sensor STIM1 via its EF-hand domain, triggering STIM1 to undergo conformational changes, oligomerization, and translocation to ER-plasma membrane (ER-PM) junctions, where it accumulates as puncta. Through its SOAR/CAD domain, STIM1 directly interacts with and activates the hexameric Orai1 channel at the plasma membrane, mediating Ca^2+^ influx (i.e. SOCE). The subsequent elevation of cytosolic Ca^2+^ concentration activates multiple downstream signaling pathways, including calcineurin-mediated NFAT dephosphorylation and nuclear translocation, activation of Ca^2+^/calmodulin-dependent protein kinase II (CaMKII), ERK1/2 signaling cascades, and the NF-κB pathway. These signals ultimately regulate gene transcription involved in pathophysiological processes such as cell proliferation, hypertrophy, migration, and inflammation. Created with BioGDP.com [29].

The overall SOCE signaling cascade is illustrated in Figure 1. The STIM family comprises STIM1 and STIM2, which act as ER Ca^2+^ sensors to gate plasma membrane Orai channels. STIM1 primarily mediates sustained Ca^2+^ plateau signals, whereas STIM2 fine‑tunes basal Ca^2+^ oscillations [30,31].

#### STIM1 and its isoforms

**Figure 2.**
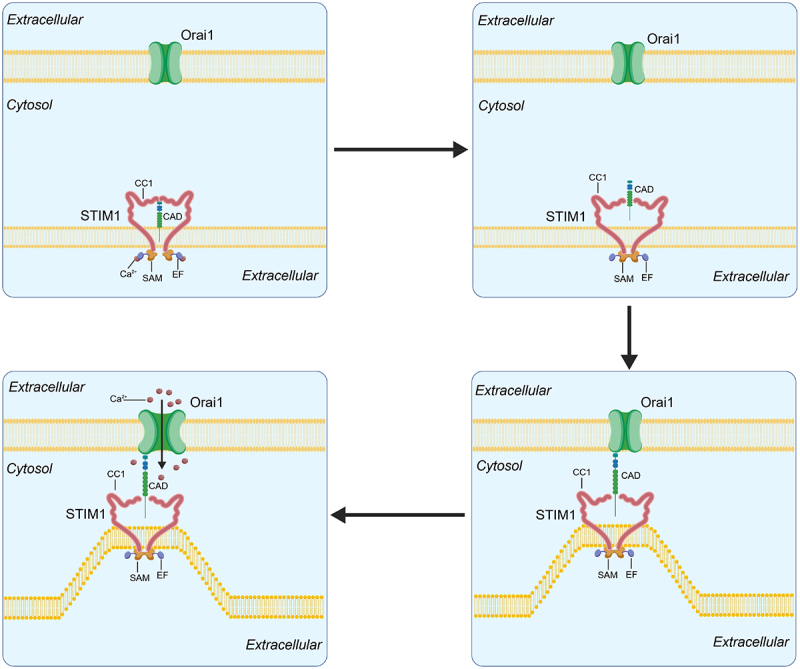
Schematic illustration of STIM1‑Orai1 coupling in store-operated Ca^2+^ entry. In the resting state (left), ER Ca^2+^ is replete, the EF-hand domain of STIM1 binds Ca^2+^, and the CAD/SOAR region is auto-inhibited by the CC1 “brake,” while Orai1 channels remain closed. Upon ER Ca^2+^ depletion (middle), Ca^2+^ dissociates from the EF-hand, triggering a conformational change of STIM1. The protein then oligomerizes and translocates to ER-plasma membrane (ER-PM) junctions, where its polybasic region anchors to the plasma membrane. In the active state (right), the released CAD/SOAR domain protrudes to capture the C-terminus of Orai1, and the resulting conformational pull opens the Orai1 pore, allowing Ca^2+^ influx. Created with BioGDP.com [29].

STIM1 is a single‑pass ER membrane protein. Its N‑terminal EF‑hand senses luminal Ca^2+^; upon store depletion, Ca^2+^ dissociation triggers conformational changes, oligomerization, and redistribution to ER plasma membrane (PM) junctions. The released CAD/SOAR domain directly gates Orai1 (Figure 2), while the polybasic C‑terminus anchors STIM1 to the PM [32,33]. Post‑translational modifications dynamically modulate STIM1 activity [34–36].

STIM1 targeting to the PM and clustering at ER‑PM junctions depend on the interaction between its C‑terminal polybasic domain and plasma membrane PI4P; the SOAR domain directly binds PI4P, and the C‑terminal lipid‑binding domain also interacts with PI(4,5)P_2_, recruiting STIM1/2 independently of Orai1 [37–39]. Lipid transfer proteins modulate phosphoinositide dynamics to fine‑tune STIM localization [40].

Alternative splicing generates functionally distinct STIM1 isoforms. STIM1L, enriched in heart and skeletal muscle, contains an actin‑binding domain (ABD) that pre‑assembles with Orai1 and TRPC1/4, enabling rapid SOCE activation [41–43]. STIM1A suppresses SOCE but promotes nuclear factor of activated T-cells (NFAT) nuclear translocation [44], while neuronal STIM1B delays SOCE activation and reduces slow Ca^2+^‑dependent inactivation [45].

#### STIM2 and its isoforms

STIM2 exhibits higher Ca^2+^ sensitivity than STIM1 and mediates modest SOCE to maintain basal Ca^2+^ homeostasis, while its SOAR domain displays weaker Orai1 coupling efficiency [46–48]. STIM2 function is also redox‑regulated via C313 sulfonation [49].

The STIM2 gene gives rise to multiple functional isoforms. The canonical STIM2.2 (STIM2α) acts as an activator sensitive to mild ER Ca^2+^ depletion, maintaining basal Ca^2+^ homeostasis [50]. In contrast, STIM2.1 (STIM2β), identified independently by two groups [17,18], contains an eight‑residue insertion in the SOAR/CAD domain that disrupts Orai1 binding, rendering it unable to activate SOCE. Instead, it exerts a dominant‑negative effect by heterodimerizing with STIM1 or STIM2.2. A third variant, STIM2.3 (also known as STIM2γ), is a C-terminally truncated isoform generated by alternative splicing, expressed predominantly in the brains of Old World primates. Unlike STIM2.1, STIM2.3 enhances store-operated Ca^2+^ entry, an effect attributed to the loss of an inhibitory C-terminal region rather than the splice-specific insert [51]. These variants regulate Orai1‑, TRPC1‑, and TRPC4‑mediated SOCE, as well as mitochondrial Ca^2+^ homeostasis in cardiomyocytes. Thus, STIM2 exerts a bidirectional regulatory network, with the relative expression of STIM2.2 and STIM2.1 fine‑tuning SOCE in a cell‑type‑specific manner [17,18,42].

### Orai protein family

The Orai family (Orai1, Orai2, Orai3) constitutes the pore‑forming subunits of CRAC channels, with distinct gating, pharmacological, and pathophysiological properties.

#### Orai1

Orai1 is the principal CRAC channel subunit. Its hexameric assembly is firmly established by multiple high‑resolution structural studies: the 3.35 Å crystal structure of Drosophila Orai [52], analyses of constitutively active H206A and P288L mutants [53–55], and cryo‑EM of human Orai1 [56] all demonstrate a hexameric pore architecture. Although pentameric assemblies have been observed in Orai1‑SOAR fusion proteins, this likely arises from the fusion construct or lipid environment [57]. Orai1 contains four transmembrane segments (TM1–TM4), with residue E106 in the pore helix determining Ca^2+^ selectivity. Channel gating involves hydrophobic residues (F99, V102) and strictly depends on STIM1 binding to Orai1’s cytosolic N‑ and C‑terminal domains [25,58].

#### Orai2

Orai2 is highly homologous to Orai1 but lacks its N‑glycosylation site and can form homo‑ or hetero‑multimers [16,59]. It mediates SOCE but negatively modulates Orai1‑mediated currents through faster Ca^2+^‑dependent inactivation [60,61]. Pharmacologically, Synta66 inhibits Orai1 but enhances Orai2, whereas BTP2 and GSK‑7975A inhibit both; high 2‑APB only mildly inhibits Orai2 [16,62].

#### Orai3

Orai3 is a mammal‑specific Orai member, sharing ~57% sequence identity with Orai1 and featuring a shorter N‑terminus and an elongated extracellular loop [60,63]. Functionally, Orai3 exhibits slow activation kinetics but markedly stronger Ca^2+^‑dependent fast inactivation than Orai1 and Orai2 [16]. Pharmacologically, low‑dose 2‑APB (<10 μM) potentiates Orai3 currents, whereas high concentrations induce STIM1‑independent pore dilation [64,65]. Orai3 can assemble with Orai1 into heteromeric pentamers to form arachidonic acid‑regulated (ARC) channels, activated independently of store depletion [23,66]. Notably, the pronounced fast inactivation of Orai3 may fine‑tune Ca^2+^ influx duration and amplitude. In cardiovascular pathophysiology, this property could modulate vascular smooth muscle cell (VSMC) proliferation, endothelial dysfunction, and cardiac excitability; Orai3 upregulation may serve as an adaptive mechanism under oxidative stress [67]. Thus, Orai3 gating kinetics shape Ca^2+^ signals in the cardiovascular system, and its dysregulation may contribute to vascular remodeling and cardiac disorders.

## The role of SOCE in cardiovascular diseases

### Auxiliary regulators of SOCE in the cardiovascular system

The canonical SOCE pathway centers on the coupling between the ER/SR Ca^2+^ sensors STIM1 (and STIM2) and the plasma membrane Orai1 channels [12,13,68]. However, recent studies have revealed that SOCE activity is also finely modulated by multiple accessory proteins. Although these proteins do not form the channel pore per se, they profoundly influence SOCE amplitude, kinetics, and downstream functions through protein–protein interactions, membrane trafficking regulation, or expression-level modulation. Among them, TRPC1, as a nonselective cation channel, can be directly gated by STIM1 via electrostatic interaction involving the STIM1 C-terminal polybasic lysine-rich domain (K684,685) and a conserved negatively charged region (D639,640) in the TRPC1 C-terminus, thereby explaining how STIM1-TRPC1 coupling contributes to the mixed nature of SOCE currents [69]. In HEK293 cells, TRPC1 silencing markedly attenuates SOCE, whereas the STIM1 (K684,685E) mutation disrupts STIM1-TRPC1 association and abolishes the concentration-dependent regulation of SOCE by extracellular Ca^2+^, indicating that plasma membrane-resident STIM1 cooperates with TRPC1 through this domain to sense the extracellular Ca^2+^ milieu [19]. Tspan18 specifically interacts with Orai1 and acts as a “molecular chaperone” to promote its trafficking to the endothelial cell surface and/or clustering; Tspan18 knockdown reduces Orai1 surface expression by ~70% and agonist-induced Ca^2+^ signaling by 55–70% [20,70], suggesting that Tspan18 serves as a “membrane localization accessory subunit” for Orai1 in a cell‑type-specific manner. Furthermore, the actin-binding protein TPM3 has recently been identified as a novel interacting partner of STIM1—RNA-seq data reveal that Tpm3 and Stim1 exhibit highly correlated transcriptomic expression across multiple rat organs, co-immunoprecipitation confirms their physical association in vascular smooth muscle cells, and knockdown of either gene significantly suppresses SOCE and SOCE-dependent vasoconstriction [21]. Collectively, these findings indicate that SOCE is not accomplished by the STIM/Orai core binary pathway alone, but rather operates within a complex regulatory network involving accessory proteins such as TRPC1, Tspan18, and TPM3. These proteins participate in SOCE fine-tuning at distinct levels – channel composition, membrane trafficking, and protein–protein interactions – in a cell‑type-specific manner, providing important molecular insights into the functional diversity of SOCE in cardiovascular physiology and pathology. As another member of the STIM family, the position of STIM2 and its splice variants within this regulatory network will be the focus of the subsequent discussion in this chapter.

### Hypertension

Hypertension is characterized by vascular injury and target organ damage, closely linked to dysregulated calcium signaling. Accumulating evidence establishes that STIM1/Orai1 mediated store operated calcium entry (SOCE) is a critical mechanism underlying vascular dysfunction in hypertension.

In hypertensive models, aortic STIM1/Orai1 expression and activity are markedly upregulated, enhancing Ca^2+^ influx and potentiating vasoconstriction. Pharmacological CRAC channel inhibition or neutralizing antibody blockade effectively ameliorates vascular hyperresponsiveness [71]. At the molecular level, vasopressin upregulates STIM1 and Orai1 in vascular smooth muscle cells (VSMCs) via the V1a receptor, enhancing SOCE and activating downstream osteogenic pathways to accelerate vascular calcification, effects abrogated by specific inhibitors [72]. Additionally, TPM3 interacts with STIM1 to modulate VSMC contraction and SOCE, contributing to high‑salt diet‑induced hypertension [21].

Beyond vasoconstriction and calcification, STIM1/Orai1‑SOCE drives VSMC proliferation and migration, processes pivotal in hypertensive vascular remodeling and neointima formation. Knockdown of STIM1 suppresses human coronary artery smooth muscle cell proliferation and reduces neointima formation after carotid artery injury in rats, via activation of the calcineurin (CaN)/NFAT pathway [73,74]. This spatial coupling paradigm is conceptually analogous to AKAP79/150, which assembles PKA, CaN, and PKC within nanodomains of L‑type Ca^2+^ channels to markedly enhance CaN/NFAT signaling efficiency [75–77]. In the context of Orai1-mediated SOCE, recent biochemical and functional evidence has directly demonstrated that AKAP79 physically interacts with the Orai1 N-terminus via the AKAR motif (residues 39–59), anchoring both calcineurin and NFAT1 in close proximity to the channel pore, thereby enabling local Ca^2+^ entry to activate the tethered transcription factor within the same nanodomain [78].

Therapeutic strategies targeting SOCE carry potential risks. Chronic systemic inhibition may paradoxically enhance compensatory sympathetic activity, predisposing patients to cardiovascular complications such as hypertension [79]. Moreover, hypertension upregulates STIM1 in VSMCs, inducing endoplasmic reticulum stress and activating TGF‑β and NADPH oxidase pathways, thereby impairing vascular function. VSMC‑specific knockout of STIM1 or the ER stress factor CHOP effectively alleviates oxidative stress, restores eNOS activity, and improves vasorelaxation [80]. STIM1 also plays a pivotal role in hypertension‑mediated kidney injury [81]. Collectively, while targeting the STIM1/Orai1 pathway holds therapeutic promise, its complex pathophysiological roles necessitate highly specific targeting strategies to achieve efficacy while mitigating the risks associated with systemic inhibition.

### Atherosclerosis

Atherosclerosis is a multifactorial vascular disease involving endothelial cells, smooth muscle cells, macrophages, and immune cells, all of which are regulated by STIM/Orai‑mediated SOCE [82].

Endothelial barrier dysfunction is not merely a contributing factor but the critical initiating event in atherogenesis, and is directly driven by STIM1/Orai1‑mediated SOCE. The pro‑inflammatory cytokine HMGB1 activates SOCE via STIM1/Orai1 in human vascular endothelial cells, leading to Src phosphorylation, VE‑cadherin downregulation, gap formation, and increased permeability – effects reversed by SOCE inhibitors or siRNA knockdown [83]. High glucose upregulates ORAI1‑3 and enhances SOCE, promoting coronary endothelial proliferation and migration, partly through complexes with IGFBP3 [84]. Moreover, ORAI1 physically interacts with VE‑cadherin, inducing PKCα‑dependent phosphorylation at Tyr731 to disrupt adherens junctions and increase permeability [85]. Thus, STIM1/Orai1‑mediated SOCE directly contributes to endothelial hyperpermeability in early atherogenesis. Following this initial endothelial breach, subsequent pathological cascades – including foam cell formation, VSMC phenotypic switching, and inflammatory recruitment – are sequentially engaged.

Macrophage foam cell formation represents the next key step in lesion progression. Activation of P2Y6R induces ER Ca^2+^ depletion via IP_3_, triggering SOCE and promoting dissociation of STIM1 from calreticulin (CALR). Released CALR enhances post‑translational modifications of scavenger receptor A, thereby accelerating ox‑LDL uptake and foam cell formation [86]. In parallel, ox‑LDL activates ORAI1 in macrophages, upregulating scavenger receptor A via calcineurin‑JNK/p38 signaling, and in vivo ORAI1 knockdown or SKF96365 reduces plaque area in ApoE^−^/^−^ mice [87].

VSMC phenotypic switching, proliferation, and migration are also critically regulated by SOCE. ox‑LDL upregulates STIM1/ORAI1 in VSMCs, promoting proliferation, migration, and invasion [88]. In hypertension, ORAI1 drives VSMC switching from a contractile to a synthetic phenotype via calcineurin‑NFAT [89]. After vascular injury, ORAI1 and ORAI3 are essential for neointima formation; ORAI1/3 heteromers can be activated by LTC_4_ in a store‑independent manner (ARC current), promoting VSMC proliferation [82,90].

In the immune-inflammatory dimension, STIM1/ORAI1‑SOCE is central to T‑cell, B‑cell, NK‑cell, and neutrophil function [82]. ORAI1 also mediates neutrophil polarization and arrest at inflamed endothelium, contributing to leukocyte recruitment and vascular inflammation [91].

Beyond these cellular events, SOCE also influences lipid metabolism and plaque stability. Endothelial progenitor cells (EPCs) from atherosclerotic mice show reduced SOCE, STIM1, and ORAI1, and SOCE inhibition further impairs EPC proliferation and migration [92]. In contrast, ox‑LDL induces EPC autophagy via SOCE‑CaMKK2‑mTOR, serving a protective role [93]. The CircUSP9X/miR‑599/STIM1 axis promotes ox‑LDL‑induced VSMC proliferation and migration, possibly in a stage‑dependent manner [94]. STIM1 deficiency alleviates lesions and modulates ORAI1 and phenotypic switching factors in vivo [88].

In summary, STIM/Orai‑SOCE participates in multiple aspects of atherosclerosis – from the initial endothelial barrier breach to macrophage foam cell formation, VSMC phenotypic modulation, immune inflammation, and plaque stability. Targeting specific ORAI isoforms or cell‑specific interventions holds therapeutic promise, yet the stage‑ and cell‑type‑dependent nature of SOCE function calls for precise spatiotemporal targeting strategies [82].

### Pulmonary arterial hypertension

Multiple signaling pathways and stress stimuli contribute to pulmonary arterial hypertension (PAH) pathogenesis by modulating store‑operated calcium entry. Ogawa et al. demonstrated that PDGF upregulates STIM1 and Orai1 expression via the Akt/mTOR axis, enhancing SOCE and promoting pulmonary arterial smooth muscle cell (PASMC) proliferation; pathway inhibition reversed these effects [95]. Hou et al. reported that chronic hypoxia upregulates STIM1, activating the SOC/Ca^2+^/NFAT pathway to induce PASMC proliferation and pulmonary vascular remodeling; STIM1 silencing abrogated this cascade [96]. Fernandez et al. further revealed that upregulation of STIM2, TRPC6, and Orai2 drives the phenotypic switch of PASMCs from a contractile to a proliferative state; suppression of these proteins attenuated SOCE, highlighting their therapeutic potential [97]. The roles of STIM and Orai proteins in PAH and associated complications were systematically reviewed by Rode et al. [98].

More recently, Masson et al. provided the first proof‑of‑concept for Orai1 inhibitors as a therapeutic strategy in PAH. In human PASMCs from PAH patients, Orai1 expression and SOCE amplitude are upregulated, coordinately driven by MEK1/2, NFAT, and NF‑κB signaling, as well as KCNK3 downregulation. Orai1 overactivity drives excessive proliferation, migration, apoptosis resistance, mitochondrial Ca^2+^ overload, and calcineurin activation – all reversed by Orai1 inhibitors (BTP2, JPIII, 5J4) or siRNA knockdown. In three rat models of PAH (chronic hypoxia, monocrotaline, and Sugen/hypoxia), Orai1 inhibition significantly reduced right ventricular systolic pressure, pulmonary vascular remodeling, and right ventricular fibrosis. Given the existing safety profile of Orai1 inhibitors in human phase I/II trials, the authors proposed that Orai1 blockade therapy warrants clinical evaluation in PAH patients [99].

Collectively, these studies demonstrate that SOCE mediated by STIM and Orai proteins – particularly the STIM1/Orai1 axis – plays a central role in PAH pathogenesis by driving PASMC proliferation, phenotypic switching, and vascular remodeling. The growing body of evidence positions the STIM/Orai‑SOCE pathway as a promising therapeutic target for PAH, warranting further translational investigation.

### Myocardial hypertrophy

In cardiac pathophysiology, store‑operated calcium entry (SOCE) mediated by STIM, Orai, and TRPC families constitutes a central signaling hub that regulates myocardial hypertrophy, fibrosis, and functional derangement. Although Orai1 plays a negligible role in excitation–contraction coupling under physiological conditions, its dysregulation under pathological states such as pressure overload markedly exacerbates cardiac dysfunction [24].

STIM1 and Orai1 coordinately mediate SOCE in cardiomyocytes, but their downstream effects diverge. Knockdown of Orai1 suppresses pathological hypertrophy by inhibiting the calcineurin pathway, whereas STIM1 knockdown exerts antihypertrophic effects primarily through inhibition of Ca^2+^/calmodulin-dependent protein kinase II (CaMKII) and ERK1/2 signaling. Notably, knockdown of either component fully abrogates phenylephrine‑induced cardiac hypertrophy, underscoring their indispensable roles [100]. Regarding STIM1 expression, while some early in vitro studies suggested that its basal level is sufficient to drive hypertrophy [101], multiple independent in vivo models – including pressure overload, Ang II infusion, and aldosterone stimulation – have consistently demonstrated significant STIM1 upregulation, indicating model‑ and context‑dependent regulation [24,80,102,103].

Beyond the canonical STIM1‑Orai1 axis, additional regulatory layers exist. STIM2.1 negatively regulates Orai1‑SOCE via mitochondria‑associated ER membrane (MAM) complexes, limiting mitochondrial Ca^2+^ uptake. In heart failure, reduced STIM2.1 expression relieves this inhibition, leading to mitochondrial Ca^2+^ overload and metabolic disturbances, thereby promoting ventricular remodeling and dysfunction [104,105]. Moreover, both STIM1 and its long splice variant STIM1L are expressed in neonatal cardiomyocytes and are upregulated in pathological cardiac hypertrophy, implying a potential involvement of STIM1L in the remodeling process [106].

A critical step downstream of STIM/Orai-mediated SOCE is the transfer of Ca^2+^ from the cytosol into the mitochondrial matrix, which is governed by the mitochondrial calcium uniporter (MCU) complex [107,108]. The MCU complex is a highly regulated channel residing in the inner mitochondrial membrane, comprising the pore-forming MCU subunit, its dominant-negative paralog MCUb, the essential MCU regulator (EMRE), and the Ca^2+^‑sensing gatekeepers mitochondrial calcium uptake proteins 1, 2, and 3 (MICU1, MICU2, MICU3) [109–112]. Under physiological conditions, MICU1/2 heterodimers maintain the MCU pore closed at low cytosolic Ca^2+^ concentrations, preventing constitutive mitochondrial Ca^2+^ overload. Upon a rise in local Ca^2+^ microdomains generated by SOCE or IP_3_ receptor-mediated release, Ca^2+^ binding to MICU EF-hand domains relieves this gatekeeping, allowing rapid Ca^2+^ flux into the matrix [110,113,114]. This mitochondrial Ca^2+^ uptake serves dual functions: it acutely buffers cytosolic Ca^2+^ to sustain CRAC channel activity by preventing Ca^2+^ -dependent slow inactivation [115–117], and it activates matrix dehydrogenases to couple energy production with contractile demand [118]. Thus, the MCU complex functions not merely as a passive downstream sink but as an active rheostat that shapes the amplitude, duration, and frequency of cytosolic Ca^2+^ oscillations, thereby feeding back on SOCE itself [117,119,120].

In the context of cardiac hypertrophy and heart failure, the MCU pathway has emerged as a double-edged sword. On one hand, acute MCU-mediated mitochondrial Ca^2+^ uptake is essential for metabolic adaptation to increased workload. On the other hand, sustained or excessive mitochondrial Ca^2+^ loading, driven by chronically enhanced SOCE and/or impaired Ca^2+^ extrusion via the mitochondrial Na^+^/Ca^2+^ exchanger (NCLX), predisposes mitochondria to permeability transition pore (mPTP) opening, oxidative stress, and cell death [121–123]. In failing human hearts, alterations in the stoichiometry of MCU regulatory subunits, including increased MICU1/MCU ratios, have been observed, which may represent an adaptive attempt to gate excessive Ca^2+^ uptake but ultimately correlate with impaired contractility [124]. Conversely, in experimental models, cardiomyocyte-specific MCU overexpression has been shown to exacerbate systolic dysfunction, whereas genetic MCU ablation or its pharmacological inhibition has provided protection against ischemia/reperfusion injury by limiting Ca^2+^ overload [121,125,126]. Importantly, the role of MCU in hypertrophy is context-dependent: in pressure overload models, MCU activity may sustain the bioenergetic demand of hypertrophic growth, but its chronic overactivation contributes to the transition from compensated hypertrophy to heart failure.

A central and unresolved question is whether MCU knockdown (or knockout) decreases or enhances SOCE. The literature is divided. Several studies using acute siRNA-mediated MCU knockdown in various cell types, including RBL mast cells and HeLa cells, reported that MCU deficiency impairs mitochondrial Ca^2+^ buffering, leading to accelerated Ca^2+^-dependent inactivation of CRAC channels and consequently reduced SOCE [117,119,127–130]. By contrast, two independent studies using CRISPR/Cas9-mediated MCU knockout in multiple cell lines and primary T cells reported that MCU deletion paradoxically enhances cytosolic Ca^2+^ signals following store depletion, owing to slowed Ca^2+^ clearance and/or compensatory transcriptional remodeling [120,131]. This paradox was recently reconciled by Bhardwaj et al. (2026), who demonstrated that in MCU-deficient RBL cells, CRAC channel activity assessed by Ba^2+^ flux is actually reduced, but the cytosolic Ca^2+^ signal is larger because plasma membrane Ca^2+^ ATPase (PMCA)‑mediated Ca^2+^ extrusion becomes saturated and slowed. Moreover, chronic MCU loss triggers a widespread adaptive transcriptomic response, including upregulation of PMCA4 and CRACR2A, which partially compensates for the loss of mitochondrial buffering. However, re-expression of wild-type MCU, but not a pore-dead mutant, fully restores normal Ca^2+^ dynamics and mitochondrial uptake, indicating that the enhanced Ca^2+^ signal is a net consequence of altered clearance rather than increased channel activity [132]. Therefore, the effect of MCU deficiency on SOCE is fundamentally different between acute knockdown (where loss of mitochondrial buffering reduces SOCE) and chronic knockout (where transcriptional adaptation and slowed clearance produce an apparent increase in cytosolic Ca^2+^). This distinction has profound implications for therapeutic targeting: acute MCU inhibition may limit Ca^2+^ overload in ischemia-reperfusion, whereas chronic MCU ablation might destabilize Ca^2+^ homeostasis through maladaptive transcriptional reprogramming and should be approached with caution.

The molecular composition of SOCE exhibits significant heterogeneity. In pathological cardiac hypertrophy, Orai3 supplants Orai1 as the predominant effector of STIM1, forming store‑independent constitutively active channels and ARC. Silencing Orai3 effectively reverses the hypertrophic phenotype, confirming its indispensability [133]. TRPC channels also contribute to SOCE in cardiomyocytes [134]. SOCE activity is subject to precise post‑translational regulation; for instance, nitric oxide restricts SOCE by S‑nitrosylating STIM1, thereby preventing cardiac hypertrophy [34].

As noted previously, AKAP79/150 assembles PKA, CaN, and PKC within nanodomains of L‑type Ca^2+^ channels to enhance CaN/NFAT signaling efficiency [75–77]. In the case of Orai1, direct evidence has now demonstrated that AKAP79 physically interacts with the Orai1 N-terminus via the AKAR motif (residues 39–59) to couple local Ca^2+^ entry to NFAT1 activation in heterologous cells [78]. Nevertheless, whether this AKAP79-Orai1 coupling operates in cardiomyocytes and contributes specifically to pathological cardiac hypertrophy remains to be established. SARAF downregulation correlates with STIM1/Orai1 upregulation in pressure overload‑induced hypertrophy, and SARAF overexpression reverses these changes while attenuating hypertrophic phenotypes [102]. Notably, STIM1 exhibits distinct regulatory patterns across different models: Ang II, aldosterone, PE, and pressure overload induce STIM1 upregulation, whereas pulmonary hypertension‑induced right ventricular hypertrophy is characterized by classical STIM1 downregulation accompanied by STIM1L upregulation [135], indicating model‑ and chamber‑specific regulation.

The pathological role of SOCE extends to cardiac fibroblasts. In right ventricular hypertrophy secondary to pulmonary arterial hypertension, upregulation of Orai1 and STIM1 in cardiac fibroblasts enhances SOCE, activating ERK1/2 and CaMKII pathways and promoting fibroblast proliferation and migration, thus driving cardiac fibrosis [136].

Targeted inhibition of SOCE holds therapeutic promise but yields complex effects. Pharmacological Orai channel inhibition or dominant‑negative mutants ameliorate electrophysiological disturbances and contractile dysfunction [137]. However, in pressure overload models, Orai1 inhibition improves systolic function and Ca^2+^ handling but does not prevent the morphological progression of cardiac hypertrophy, revealing a signaling decoupling between hypertrophic growth and functional deterioration [24]. Furthermore, cardiomyocyte‑specific Orai1 deletion in adult mice aggravates AngII‑induced hypertrophy and fibrosis, revealing a protective role of Orai1 in adult cardiomyocytes that contrasts with its pathogenic role in neonatal and certain pathological settings [138]. Collectively, these findings establish the SOCE network as a critical therapeutic target, yet underscore the necessity for highly specific intervention strategies tailored to distinct molecular components, cell types, and disease stages.

### Vascular embolic disorders

SOCE serves as a core signaling pathway governing platelet activation and vascular remodeling, with STIM1, Orai1, and their regulatory modulators emerging as critical therapeutic targets in thrombo-occlusive and vascular proliferative disorders.

In vascular smooth muscle cells (VSMCs), STIM1/Orai1-mediated SOCE drives proliferation and migration. STIM1 is markedly upregulated in proliferating VSMCs and in the neointima following vascular injury. Silencing of STIM1 effectively suppresses human coronary artery smooth muscle cell proliferation and reduces neointima formation after carotid artery injury in rats via the calcineurin/NFAT pathway [73]. Similarly, PDGF specifically activates STIM1/Orai1-dependent Ca^2+^ influx – rather than Orai2/3 or TRPC channels – through PDGFR β, promoting VSMC migration; in vivo, STIM1, Orai1, and PDGFRβ are coordinately upregulated in injured VSMCs [74].

Beyond this canonical store-operated pathway, a non-canonical activation mode exists in VSMCs. Thrombin activates a store‑independent Ca^2+^ entry pathway via intracrine leukotriene C_4_ (LTC_4_) produced downstream of receptor stimulation. Unlike PDGF, thrombin‑evoked currents require both Orai1 and Orai3 as an obligate heteromer, with STIM1 acting downstream of LTC_4_ but independent of store depletion. This LTC_4_‑regulated Orai1/3 channel is specifically upregulated in medial and neointimal VSMCs after vascular injury, and in vivo knockdown of Orai3 abrogates the currents and markedly reduces neointimal hyperplasia, offering a more selective strategy than targeting Orai1 alone [90].

In platelets, Orai1 serves as the principal Ca^2+^ channel mediating SOCE and is critically involved in thrombus formation under high shear stress. Orai1 deficiency markedly impairs SOCE, rendering mice substantially resistant to pathological thrombosis (pulmonary embolism, arterial thrombosis, and cerebral infarction) with only a mild bleeding tendency, highlighting Orai1 as a promising and safe antithrombotic target [139]. STIM1, acting as an upstream regulator, forms a critical signaling axis with Orai1 and TRPC1 that governs Ca^2+^ influx and plays a central role in thrombin- and ADP-induced platelet aggregation [140,141]. Platelet SOCE is subject to finely tuned redox regulation. Endoplasmic reticulum oxidoreductase 1α (ERO1α), localized to the dense tubular system, oxidizes specific disulfide bonds – Cys49–Cys56 and Cys875–Cys887 – within STIM1 and SERCA2, thereby promoting platelet activation, arterial thrombosis, and ischemic stroke. Genetic or pharmacological inhibition of ERO1α effectively attenuates thrombosis and stroke injury without increasing bleeding risk, underscoring the therapeutic potential of targeting upstream SOCE regulators [142]. Modulating STIM1/Orai1 interactions with specific proteins such as Tspan18 represents a potential strategy with enhanced specificity [20].

Under pathological conditions, this pathway undergoes functional remodeling. Platelets from patients with type 2 diabetes mellitus and peripheral artery disease exhibit enhanced SOCE, closely associated with upregulation of STIM1 and SERCA3 and activation of the PLC pathway. Although menthol attenuates this effect by inhibiting PLC, its efficacy is diminished in patients, underscoring the critical role of this pathway in platelet dysfunction associated with diabetes [143].

In summary, the STIM1/Orai1 signaling axis plays a multifaceted and complex role in thrombosis and vascular diseases. While direct inhibition carries potential risks, precise interventions targeting specific components (such as Orai1), upstream regulators (ERO1α), pathological isoforms or heteromers (Orai3 or Orai1/3), or protein–protein interaction interfaces offer a highly promising strategic direction for the development of novel and safe antithrombotic and anti-restenosis therapies [26,90,142,144].

**Figure 3.**
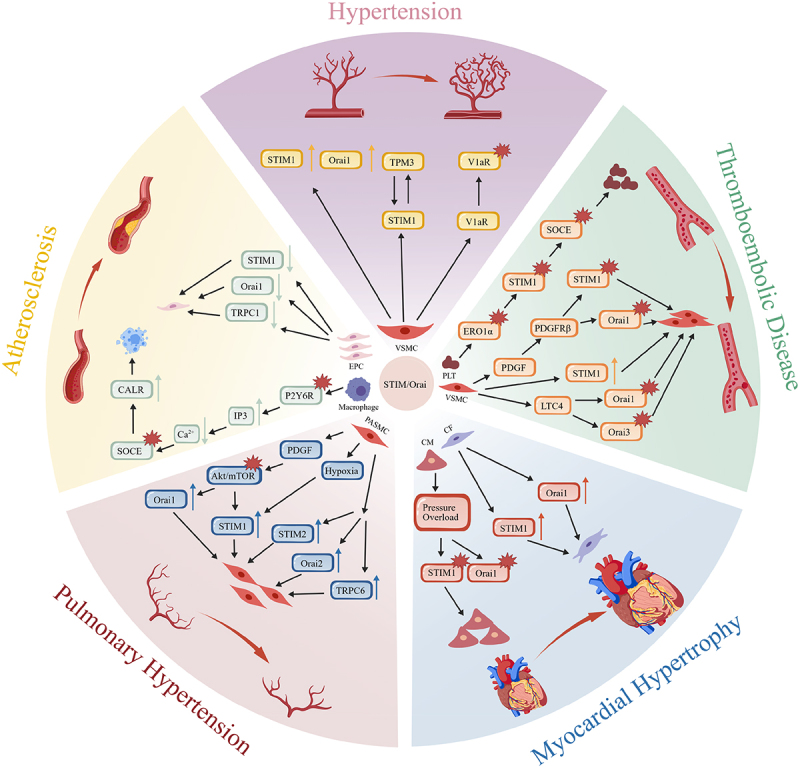
Cell type-specific roles of the STIM/Orai-mediated SOCE pathway in cardiovascular diseases. This schematic illustrates how the core SOCE machinery is differentially engaged across vascular cell types to contribute to distinct cardiovascular pathologies. In hypertension, upregulation of STIM1 and Orai1 in vascular smooth muscle cells (VSMCs), coupled with TPM3 interaction and V1a receptor activation, enhances vascular reactivity and promotes renal injury. In atherosclerosis, P2Y6R activation in macrophages triggers ER Ca^2+^ depletion, promoting STIM1-CALR dissociation and accelerating foam cell formation; conversely, downregulation of STIM1, Orai1, and TRPC1 in endothelial progenitor cells (EPCs) impairs SOCE-driven proliferation and migration, reducing vascular repair capacity. In pulmonary arterial hypertension, the PDGF/Akt/mTOR pathway and hypoxia upregulate STIM1/ Orai1 along with STIM2, TRPC6, and Orai2 in pulmonary arterial smooth muscle cells (PASMCs), driving a proliferative phenotypic switch that leads to vascular remodeling and elevated pressure. In cardiac hypertrophy, pressure overload activates STIM1/ Orai1 signaling in cardiomyocytes, where Orai3 replaces Orai1 as the primary effector under pathological conditions; concurrent upregulation of STIM1/ Orai1 in cardiac fibroblasts enhances SOCE to promote proliferation and migration, collectively contributing to hypertrophy, electrophysiological abnormalities, and fibrosis. In thromboembolic diseases, Orai1 serves as the primary SOC channel in platelets, where its activity is regulated by ERO1α-mediated oxidative modification of STIM1 (Cys49-Cys56 disulfide bond), promoting arterial thrombosis and ischemic stroke. Following vascular injury, upregulated STIM1 in VSMCs is activated by PDGF via PDGFRβ, while thrombin-induced LTC4 generation activates store-independent Orai1/3 channels (LRC), jointly driving neointimal hyperplasia and restenosis. Created with BioGDP.com [29].

In the context of cardiovascular diseases, the SOCE network functions as a highly heterogeneous and spatiotemporally regulated signaling hub, engaging distinct molecular components, cell types, and activation modalities across different pathological settings (Figure 3 and Table 1). In hypertension, upregulated STIM1/Orai1 in VSMCs enhances vasoconstriction and drives vascular remodeling; in atherosclerosis, SOCE exerts opposing effects – promoting foam cell formation in macrophages while impairing endothelial progenitor cell function; in pulmonary arterial hypertension, upregulated Orai1 and its interacting partners drive PASMC proliferation and phenotypic switching; in cardiac hypertrophy, the Orai3‑STIM1 pathway supplants the canonical Orai1‑STIM1 axis to mediate pathological remodeling; and in thromboembolic diseases, Orai1‑mediated SOCE in platelets and Orai1/3 heteromeric channels in VSMCs contribute to thrombosis and neointimal hyperplasia, respectively. Accordingly, future therapeutic strategies targeting this pathway must precisely account for specific components (e.g. Orai3), distinct cell types (e.g. macrophages vs. VSMCs), disease contexts (e.g. diabetes), and even particular activation modalities (e.g. store‑independent LTC_4_‑regulated Orai1/3 channels) to maximize therapeutic efficacy while minimizing potential systemic off‑target effects.

**Table 1.** Cell-type-specific downstream effects following activation of the STIM1/Orai1 axis.

Cell types	Main downstream pathways	Physiological/pathological effect
VSMCs	CaMKII、NFAT、ERK1\2	Contraction、Proliferation、Migration
Cardiomyocyte	CaN\NFAT、CaMKII	Cardiac hypertrophy
Platelet	GPCR signaling pathway	Activation、Aggregation
Macrophage	CALR-SRA signaling pathway	Foam cell formation
Endothelial cell	eNOS	Vasodiation
Fibroblast	ERK1\2、CaMKII	Proliferation、Migration、fibrosis

## The potential of the STIM/Orai system as a therapeutic target in cardiovascular disease

### Pharmacological tools targeting STIM/Orai signaling

Several small‑molecule inhibitors have been developed to modulate STIM/Orai‑mediated SOCE (Table 2). BTP2 inhibits CRAC channels with an IC_50_ of 10‑100 nM but has off‑target effects on TRPM4, TRPC3, and TRPC5 [145]. GSK‑7975A inhibits Orai1 and Orai3 with an IC_50_ of ~4 µM [146]. JPIII, a Synta66 analog, shows nanomolar potency and efficacy in cardiac hypertrophy and pulmonary hypertension models [24,99]. Synta‑66 exhibits IC_50_ ~26 nM in vascular cells. 2‑APB is the most widely used but most promiscuous compound, and its use should be limited to a research tool rather than a therapeutic candidate [147]. Notably, none of these compounds were originally developed for cardiovascular applications – most emerged from immunology research – and all suffer from insufficient isoform selectivity, poor pharmacokinetics, and off‑target toxicities, highlighting the urgent need for cardiovascular‑dedicated SOCE inhibitors.

**Table 2.** Pharmacological modulators of STIM/Orai-mediated SOCE.

Drug	Target	Main action
BTP2	CRAC channels (Orai1/Orai2)	Inhibits CRAC (IC_50_ 10–100 nM); attenuates hypertrophy and vascular remodeling (off-target on TRPM4/TRPC3/5).
GSK-7975A	Orai1 and Orai3	Blocks Orai1/3 (~4 µM); originally for immune use.
JPIII	Orai1 (nanomolar potency)	Potent Orai1 blocker; improves systolic function and Ca^2+^ handling in hypertrophy/PAH.
Synta-66	Orai1	Selective Orai1 inhibitor (IC_50_ ~26 nM in vascular cells).
2-APB	Nonselective(Orai1/2/3,TRPC,IP_3_R)	Biphasic Orai modulator (low µM potentiates Orai3, high inhibits); research tool only.
SKF96365	Nonselective SOCE inhibitor	Nonselective SOCE blocker; reduces foam cells and plaque in ApoE^−^/^−^ mice.
5J4	Orai1	Orai1 inhibitor; attenuates pulmonary vascular remodeling and fibrosis in PAH rats.

Orai isoforms display distinct pharmacological profiles: BTP2 and GSK‑7975A abrogate Orai1 and Orai2 while only partially inhibiting Orai3, whereas Synta66 inhibits Orai1 but potentiates Orai2 [62]. The ARC, which requires both Orai1 and Orai3 [23], adds further complexity to isoform‑selective targeting. Collectively, the lack of isoform‑selective inhibitors – particularly Orai3‑selective compounds that would be desirable for cardiac hypertrophy – represents a major bottleneck for therapeutic translation.

### Preclinical evidence in cardiovascular disease models

Cardiac Hypertrophy and Heart Failure. JPIII preserved systolic function and normalized Ca^2+^ handling in pressure‑overload mice without preventing morphological hypertrophy [24]. Phenylephrine-induced hypertrophy involves CaMKIIδ upregulation, which enhances SOCE; accordingly, the SOCE blocker BTP2 attenuated this hypertrophic response by inhibiting the downstream calcium influx [103]. Orai3 emerges as the predominant STIM1 effector during pathological hypertrophy, suggesting that Orai3‑selective inhibition may be more desirable than pan‑Orai blockade [133]. STIM1‑dependent SOCE, operating through calcineurin‑NFAT, is required for pathological hypertrophy; STIM1 is re‑activated under stress, including the STIM1L splice variant [106]. The endogenous inhibitor SARAF negatively modulates SOCE; its overexpression attenuated hypertrophy by suppressing STIM1/Orai1 upregulation without affecting basal levels [102]. However, cardiomyocyte‑specific Orai1 deletion in adult mice aggravated AngII‑induced hypertrophy and fibrosis, revealing a protective role of Orai1 in adult cardiomyocytes that complicates therapeutic strategies [138].

Atherosclerosis and Vascular Remodeling. Orai1 mediates oxLDL‑induced Ca^2+^ entry in macrophages, driving SR‑A expression and foam cell formation via calcineurin‑ASK1‑JNK/p38 signaling. In vivo Orai1 knockdown or SKF96365 treatment inhibited plaque development in ApoE^−^/^−^ mice [87]. In VSMCs, STIM1/Orai1 signaling drives proliferation, migration, and phenotypic switching. The circUSP9X/miR‑599/STIM1 axis promotes oxLDL‑induced VSMC proliferation [94], while Sarsasapogenin blocks these effects via STIM1 suppression [148]. STIM1 mediates AngII‑induced Egr‑1 expression via SOCE, CaM/CaMKII and ERK1/2/CREB pathways [149]. Smooth muscle‑specific STIM1 deletion protected against hypertension‑induced cardiac and vascular dysfunction through ER stress abrogation [80]. In SHR, Orai1 upregulation in coronary arteries promotes VSMC phenotypic switching via calcineurin‑NFAT [89]. In endothelial cells, SOCE regulates EPC function; high glucose upregulates Orai1‑3, forming complexes with IGFBP3 or VE‑cadherin that regulate permeability and migration [84,85].

Pulmonary arterial hypertension . BTP2, JPIII, and 5J4 attenuated vascular remodeling and improved right ventricular function in rat models [99]. BTP2 reduced [Ca^2+^]_i_ transients and SR Ca^2+^ load in hypertrophied RV myocytes. Aldosterone upregulates TRPC1, TRPC5, and STIM1 via MR activation, enhancing SOCE [135]. Given the existing safety profile of Orai1 inhibitors in human phase I/II trials (originally conducted for autoimmune indications), Orai1 blockade therapy warrants clinical evaluation in PAH patients [99].

Thrombosis and Inflammation. Orai1‑deficient mice are resistant to pathological thrombosis with a mild bleeding tendency [139]. Orai1 mediates neutrophil arrest and polarization under shear flow, positioning it as a key regulator of innate immune recruitment [91]. STIM1 silencing suppresses VSMC proliferation and neointima formation [73]. Store‑independent Orai1/3 channels activated by intracrine LTC4 also contribute to neointimal hyperplasia, suggesting that targeting Orai3 or the Orai1/3 heteromer may offer a more selective anti‑restenosis strategy than targeting Orai1 alone [90].

### Translational barriers and future strategies

Despite promising preclinical evidence, translating SOCE‑targeted therapies into clinical cardiovascular applications faces five major barriers – isoform selectivity, pharmacokinetics, tissue‑specific delivery, disease‑stage‑dependent effects, and lack of validated biomarkers – each manifesting differently across disease contexts. Isoform selectivity is complicated by Orai1’s protective role in adult cardiomyocytes versus its pathogenic role in hypertrophy [106,138], while Orai3 supplants Orai1 as the predominant effector in cardiac hypertrophy, making Orai3‑selective inhibition preferable [133]; in thromboembolic diseases, Orai1 is the principal platelet SOC channel [139], whereas Orai1/3 heteromers drive neointimal hyperplasia [90], demanding distinct strategies that are not yet available. Pharmacokinetic limitations, particularly poor oral bioavailability of existing inhibitors (e.g. BTP2, Synta‑66), hinder chronic disease applications. Systemic inhibition may cause unintended effects, including enhanced sympathetic activity [79] and renal injury in hypertension [81], necessitating cell‑type‑specific delivery systems such as lipid nanoparticles [150]. The therapeutic window also varies with disease stage: Orai1 inhibition improves function without preventing morphological hypertrophy [24]; SOCE exerts opposing effects in atherosclerosis depending on lesion stage [92,93]; whereas in pulmonary arterial hypertension, consistent efficacy across models suggests less stage‑dependency [99]. Finally, the absence of validated biomarkers complicates patient stratification and trial design. Addressing these barriers through isoform‑selective inhibitors, improved formulations, targeted delivery, stage‑adapted strategies, and biomarker development will be essential to bridge the gap between fundamental discoveries and clinical applications.

### The amlodipine controversy: Off‑target activation or experimental artifact?

High‑profile study reported that amlodipine and other L-type Ca^2+^ channel blockers (LCCBs) activate STIM/ORAI-mediated store‑operated Ca^2+^ entry at micromolar concentrations via the STIM1 N‑terminus (residues Δ29‑46/Δ31‑40), promoting vascular remodeling and correlating with increased heart failure risk in patients [151]. A subsequent correction clarified the odds ratios and noted modest associations with nonuterine leiomyomas [152].

However, these conclusions have been robustly refuted. Amlodipine exhibits intrinsic fluorescence overlapping with fura‑2 and accumulates intracellularly, rendering fura‑2-based Ca^2+^ measurements invalid; using longer‑wavelength dyes (Cal‑520, Fluo‑4, Fura Red) that avoid this interference, no Ca^2+^ influx was detected at therapeutic (sub‑µM) concentrations [27,28]. The therapeutic unbound concentration of amlodipine in patient serum is in the low nanomolar range (0.7‑36 nM) – orders of magnitude below the µM levels used to claim CRAC activation. Furthermore, NMR spectroscopy showed that amlodipine is ~99% bound to serum proteins, reducing free concentrations in culture media from 0.5 µM to ~5 nM. Direct measurement of PBMCs from patients taking amlodipine for >15 years revealed no detectable intracellular drug accumulation. STIM1 puncta formation was induced by carbachol but not by 0.5 µM amlodipine, and ER Ca^2+^ release was only observed at concentrations ≥20 µM [28].

Meta‑analyses of RCTs and real‑world analysis of >63,000 patients on monotherapy showed that dihydropyridine CCBs are not associated with increased heart failure; rather, they are protective compared with placebo [27]. The current consensus is that amlodipine does not engage CRAC channels at clinically relevant concentrations, and the reported SOCE activation is an experimental artifact from amlodipine’s fluorescence. The recommendation to avoid LCCBs in hypertensive patients is not supported by evidence.

## Conclusion and future perspectives

SOCE, mediated by the core molecular components STIM and Orai proteins, plays a critical role in cardiovascular physiology and pathology through its regulation of intracellular calcium homeostasis. Upon depletion of endoplasmic reticulum calcium stores, STIM1 and STIM2 activate Orai channels – principally Orai1 and Orai3 – thereby initiating calcium influx that contributes to key processes such as vascular contraction, smooth muscle proliferation, platelet activation, and myocardial hypertrophy. Dysregulated SOCE has been closely linked to the pathogenesis of several major cardiovascular disorders, including hypertension, atherosclerosis, pulmonary hypertension, and thrombotic diseases.

At the molecular level, beyond the classical STIM-Orai axis, SOCE is subject to fine-tuned regulation by multiple regulatory networks. STIM2 splice variants, particularly the inhibitory STIM2.1, negatively modulate Orai1-SOCE through mitochondria-associated endoplasmic reticulum membrane complexes, playing a critical metabolic regulatory role in heart failure. Accessory proteins including TRPC1, Tspan18, and TPM3 participate in SOCE fine-tuning at distinct levels – channel composition, membrane trafficking, and protein–protein interactions. Furthermore, the “channel-scaffold-phosphatase” spatial integration paradigm exemplified by AKAP79/150 provides an important conceptual framework for understanding the efficient coupling of the SOCE-CaN-NFAT signaling axis in vascular remodeling, while the endogenous inhibitor SARAF constitutes an additional layer of negative feedback regulation. Collectively, these findings indicate that SOCE is not a simple linear transduction of the STIM/Orai core binary pathway, but rather a complex regulatory network involving multiple accessory proteins and modulatory factors in a cell‑type-specific manner.

Under pathological conditions, SOCE drives disease progression through multiple mechanisms. However, its function is not uniformly pathogenic – Orai1 exerts a protective role in adult cardiomyocytes while promoting hypertrophy in neonatal cells and certain pathological contexts, posing a significant challenge for therapeutic strategy design. The SOCE network functions as a highly heterogeneous and spatiotemporally regulated signaling hub, engaging distinct molecular components, cell types, and activation modalities across different diseases: in hypertension, upregulated STIM1/Orai1 in VSMCs enhances vasoconstriction; in atherosclerosis, SOCE promotes foam cell formation in macrophages while its downregulation in endothelial progenitor cells impairs repair capacity; in pulmonary arterial hypertension, upregulated Orai1 and its interacting partners drive PASMC proliferation; in cardiac hypertrophy, Orai3 replaces Orai1 as the primary effector of STIM1; and in thromboembolic diseases, platelet Orai1 and VSMC Orai1/3 heteromeric channels contribute to thrombosis and neointimal hyperplasia, respectively.

Pharmacological or genetic targeting of SOCE has shown therapeutic promise in preclinical animal models. However, clinical translation faces five major barriers: insufficient isoform selectivity among Orai family members, suboptimal pharmacokinetic properties, lack of tissue-specific delivery, disease stage-dependent effects, and the absence of validated biomarkers. Notably, amlodipine and other L-type calcium channel blockers do not activate CRAC channels at therapeutic concentrations; the reported SOCE activation is an experimental artifact arising from fluorescence interference, and the associated clinical recommendations lack evidentiary support. Future research should focus on: elucidating the cell-type-specific regulatory networks governing SOCE; developing highly selective Orai3 inhibitors or strategies targeting specific protein–protein interaction interfaces; optimizing the pharmacokinetic profiles of SOCE inhibitors and establishing tissue-specific delivery systems (e.g. lipid nanoparticles); integrating multi-omics approaches with artificial intelligence to guide precision intervention design; and developing biomarkers for patient stratification and efficacy monitoring. These advances are essential to bridge the gap between fundamental discoveries and clinical applications, thereby establishing SOCE as a viable therapeutic axis for cardiovascular disease.

## Data Availability

No new research data were generated during this study. The data supporting the conclusions of this study are derived from previously published, publicly available literature and are listed in the references.
